# Perceived cognitive effectiveness and performance‐based cognitive dysfunction in heart failure

**DOI:** 10.1002/alz.092594

**Published:** 2025-01-03

**Authors:** Catherine M Martin, Susan J Pressler, Miyeon Jung

**Affiliations:** ^1^ Indiana University School of Nursing, Indianapolis, IN USA; ^2^ Indiana Alzheimer Disease Center, Indianapolis, IN USA

## Abstract

**Background:**

Cognitive dysfunction occurs in approximately 40% of individuals diagnosed with heart failure (HF). This study aims to describe perceived cognitive effectiveness among patients with HF and examine associations with performance‐based measures of cognitive dysfunction.

**Method:**

Baseline data were used from a 2‐group randomized controlled trial testing a cognitive intervention to improve attention among 73 patients with HF. Data were collected by telephone interviews due to COVID‐19 pandemic from July 2020 to May 2021. Perceived cognitive effectiveness was measured by the Attentional Function Index (AFI). AFI has 13 items assessing self‐reported effectiveness of performing cognitive activities in domains of attention, working memory, and executive function on a visual analogue scale (possible range: 0 – 100 by averaging the 13‐item scores, higher = better). Performance‐based cognitive dysfunction was measured by Oral Trail Making A and B for attention and executive function, respectively (higher response time in seconds = worse). Pearson’s correlation coefficients were used to examine associations between AFI and Oral Trail Making A and B. The significance level was set at p < .05.

**Result:**

Of 73 patients HF, 56% were women, and the average age was 66 years. On average, perceived cognitive effectiveness was moderate (AFI = 68.4 ± 15.03, range = 23 ‐ 98). Only 35.6% of patients reported high perceived cognitive effectiveness (AFI total score ≥ 75). Mean Oral Trail Making A score was 9.4 seconds (SD = 4.0); Oral Trail Making B score was 38.2 seconds (SD = 17.9). Better perceived cognitive effectiveness was significantly correlated with better attention (Oral Trail Making A, r = ‐0.38, p < .001) as well as better executive function (Oral Trail Making B, r = ‐0.27, p = .021).

**Conclusion:**

Most participants with HF perceived themselves to be moderately effective in performing cognitive activities. Given the statistically significant but small to moderate correlations between subjective and objective measures of cognitive dysfunction, administering both types of measures may aid in early detection of persons at risk for developing cognitive impairment.

